# Tissue resident memory T cells inhabit the deep human conjunctiva

**DOI:** 10.1038/s41598-022-09886-3

**Published:** 2022-04-12

**Authors:** Racha Arnous, Sana Arshad, Kerrie Sandgren, Anthony L. Cunningham, Nicole Carnt, Andrew White

**Affiliations:** 1 Centre for Vision Research, The Westmead Institute for Medical Research, 176 Hawkesbury Road, Westmead, NSW 2145 Australia; 2 Centre for Virus Research, The Westmead Institute for Medical Research, 176 Hawkesbury Road, Westmead, NSW 2145 Australia; 3 School of Optometry and Vision Science, University of New South Wales, Kensington, NSW 2033 Australia; 4 Faculty of Medicine and Health, The University of Sydney, Sydney, NSW 2006 Australia; 5 Save Sight Institute, The University of Sydney, Sydney, NSW 2000 Australia

**Keywords:** Adaptive immunity, Immunological memory

## Abstract

Mucosal linings of the body, including the conjunctiva, are enriched in tissue-resident memory T cells (T_RMs_) whose defining feature is their continual tissue protection that does not rely on migration to lymphoid organs to elicit immune responses. Hitherto, conjunctival T_RMs_ have only been identified in the superficial epithelium. This work aims to develop a more complete understanding of the conjunctival immunological capacity by investigating the presence of T_RMs_ within the deeper, more stable layers of the healthy human conjunctiva. Using immunofluorescence microscopy and antibodies against CD3, CD4, CD69 and HLA-DR on bulbar conjunctival biopsies obtained from 7 healthy adults (age range = 32–77 years; females = 4), we identified CD69^+^T_RM_ subsets in all layers of the human conjunctiva: the superficial epithelium, the basal epithelium, the adenoid, and the fibrous layers. Interestingly, the adenoid layer showed significantly higher densities of both CD4 and CD8 T_RMs_ when compared to the fibrous layer and conjunctival epithelia. Additionally, CD4 T_RMs_ predominated significantly over CD8 T_RMs_ in the adenoid layer. The abundance of deep conjunctival CD69^+^T_RMs_ within the healthy human may suggest the presence of defence mechanisms capable of inducing long-term immunogenic memory. Understanding this spatial distribution of conjunctival CD69^+^T_RMs_ is essential to improving mucosal vaccine design.

## Introduction

**Figure 1:**
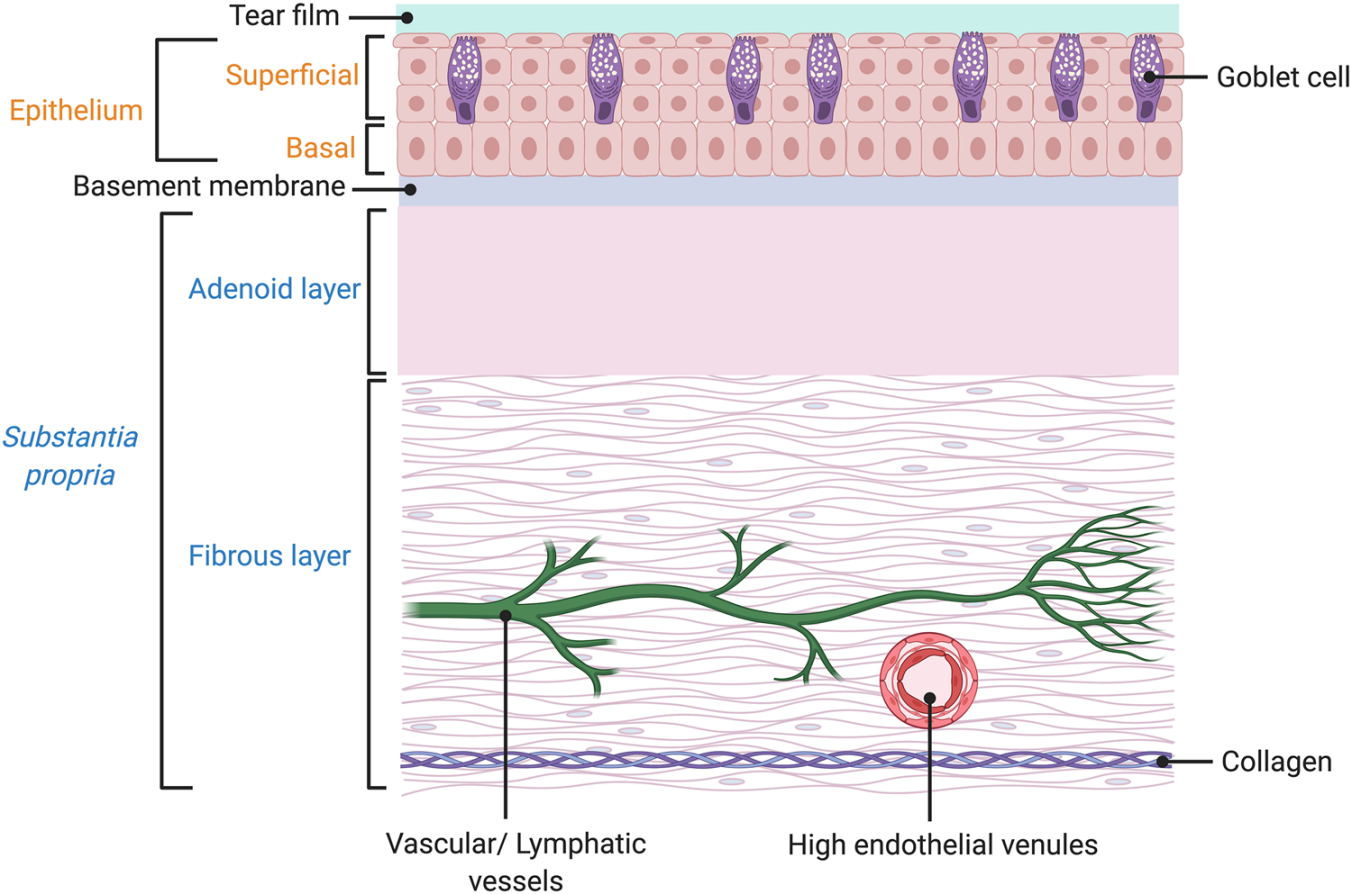
Schematic diagram showing the histological layers of the human conjunctiva. Figure created with BioRender.com.

The conjunctiva is a fragile yet complex mucosal membrane located anterior to the white sclera, beginning at the corneal limbus and extending to cover the inner surface of the eyelids^1,2^. Histologically, it comprises the superficial epithelium, the basal epithelium, the adenoid layer, and the fibrous layer^3^ (Fig. 1). The latter two layers form the loose connective tissue known as the *substantia propria*^4^*,* separated from the basal epithelium by a thin basement membrane. In this study, all conjunctival layers below the superficial epithelium are termed the deep conjunctiva and constitute the basal epithelium, adenoid and fibrous layers. The mean thickness of the healthy bulbar conjunctival epithelium has a depth of 42.4 ± 7.4 µm^5^ and varies between 2 and 5 cells, of which the deepest cell layer can be classified as the basal epithelium^4^. The mean thickness of the *substantia propria* is 197.7 ± 32.5 µm^5^, of which the adenoid layer has a thickness with the same range as the epithelium and extends from the basement membrane^6^ to the lower fibrous layer and connects with the episclera. There are no studies that have measured the thickness of the fibrous layer, however, subtracting the approximate thickness of the full epithelium from the mean thickness of the *substantia propria* gives an estimated 160 µm of fibrous tissue that was used for this study.

The conjunctiva-associated lymphoid tissue (CALT) is formed by the abundant lymphoid follicles and diffuse lymphoid tissues present in the *substantia propria*^6^. CALT can detect and present antigens and drain them into the nasal-associated lymphoid tissue (NALT) that is continuous with the lung mucosa and its bronchus-associated lymphoid tissue (BALT)^7^. This mucosal immunity, initiated by CALT, has established the conjunctival mucosa as a potential route for mucosal vaccine delivery^8^. Such eye drop administration of vaccines are already being used on poultry to control the viral respiratory and highly contagious Newcastle disease^9,10^ but are yet to be utilised for humans. Therefore, understanding conjunctival immunology and its ability to induce an effective, long-lasting immunogenic memory will be vital for the development of such vaccines that have the potential of a delivery system that is easily administrable, affordable and allows for rapid worldwide distribution.

Recently, tissue-resident memory T cells (T_RMs_) have been critical cells of interest in mucosal vaccine studies. These cells are a subtype of non-circulating memory T cells that remain localised in peripheral tissues after antigenic challenge to enhance long-term immunity and immunosurveillance upon re-exposure^11,12^. Their broad and long-lasting immune protection at mucosal surfaces, including their ability to secrete inflammatory and antiviral cytokines, and communicate with dendritic cells and macrophages^13–17^, may provide a key immune effector mechanism for mucosal vaccines. Furthermore, as contagious pathogens are most often encountered at peripheral mucosal surfaces, the continual presence of a mucosal T_RM_ population is important for pathogen control and elimination at the site of entry^18^. In contrast, tissues without a local T_RM_ population allow pathogens to multiply and circulate within the body whilst the host recruits circulating memory T cells to the site of infection^18,19^. Therefore, the induction of T_RMs_ has become a new vaccine development strategy.

A common clinical protocol for conjunctival tissue collection is ocular surface impression cytology, in which an absorbent filter paper is pressed onto the surface of the conjunctiva to remove the most superficial layer of cells^20^. Cells present within this superficial layer are perpetually exposed to friction from the blinking eyelid, and thus are sloughed off into the tears to be replaced by basal epithelial cells^21,22^. In 2017, Bose et al.^23^ used impression cytology to examine the resident populations of T cells in the healthy conjunctiva. Their flow cytometric analysis determined that the conjunctiva is protected by two T_RM_ subsets, CD69^+^CD103^−^ and CD69^+^CD103^+^ cells. However, the continual sloughing of the superficial epithelium where these T_RM_ subsets were identified, minimises their most crucial function: their long-term protection of the ocular surface. In contrast, deep conjunctival layers remain stable with their resident cell populations as they are not exposed to these frictional forces from blinking. Thus, immune cells populating the deep conjunctiva may provide the ocular surface with longer-lasting, specific immune protection. Additionally, T_RMs_ in the *substantia propria* could indicate potential communication with antigen-presenting cells that are abundant within this layer^24^ and may allow for a more robust elimination of pathogens. Nevertheless, the presence and relative proportions of T_RM_ cell subsets in the deep conjunctiva are yet to be studied in healthy humans. Such information could provide insight into the immunological response profile of the normal conjunctiva and allow for the diagnosis of abnormal profiles from inflammatory ocular diseases.

In the present study, we investigated healthy human conjunctival biopsies with complete and preserved histomorphologies of the epithelium and *substantia propria* to determine the presence and distribution of T_RM_ cell subsets in the deep conjunctival layers using immunofluorescent microscopy.

## Results

A total of 18 healthy cataract surgery patients with no underlying systemic conditions were enrolled, and all of them met the inclusion criteria. The analysis was completed on seven conjunctival biopsies from 7 healthy cataract patients (age range = 32–77 years; females = 4) (see supplementary Table S1). Excluded cases, 11 in total, had tissues demonstrating inflammation or severe folding determined by histological analysis due to their collection using an unoptimized protocol at the time. The surgical excisions preserved the entire histological anatomy of the conjunctival tissue and comprised the entire superficial and basal epithelia and the underlying loose layers of the *substantia propria*; except for one conjunctival sample where the fibrous layer was not surgically collected.

### CD69^+^ T_RM_ subsets reside in all conjunctival layers; however, CD4 T_RMs_ predominate within the adenoid

**Figure 2:**
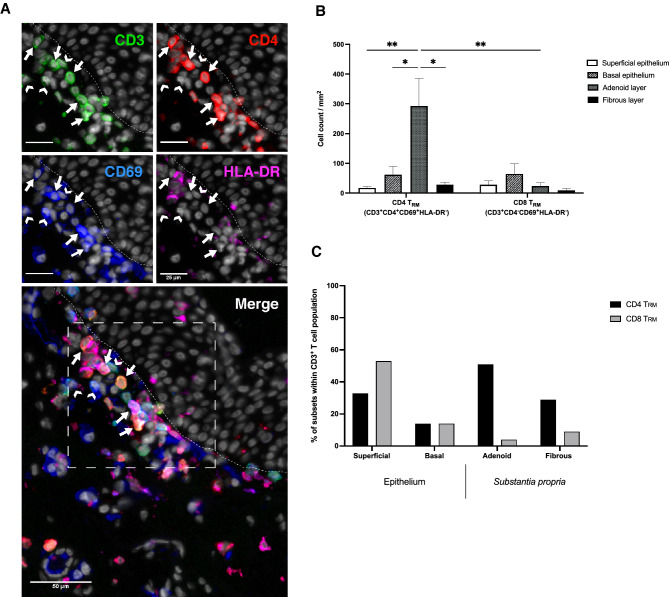
CD69^+^ T_RM_ cells localise in all layers of the healthy human conjunctiva. Human conjunctival biopsy sections were stained with CD3 (green), CD4 (red), CD69 (blue) and HLA-DR (magenta). (**A**) Representative immunofluorescent staining showing CD4 T_RMs_ (CD3^+^CD4^+^CD69^+^HLA-DR^−^: arrows) and CD8 T_RMs_ (CD3^+^CD4^-^CD69^+^HLA-DR^−^: arrowheads) in the adenoid layer below the basement membrane (dashed line). All images were taken at × 20 magnification. Panels showing individual channels have scale bars indicating 25 µm. The image of the merged channels is shown on the bottom. (**B**) Average numbers of CD4 T_RM_ and CD8 T_RM_ cell counts per mm^2^ are displayed in the different layers of the conjunctiva: superficial epithelium, basal epithelium, adenoid layer and fibrous layer. Values are given as mean ± S.E.M. Statistical significance was assessed by a two-way-ANOVA, post hoc Tukey HSD multiple comparison test; *n* = 7 (except fibrous layer = 6). Asterisks indicate significance values: *P < 0.05; **P < 0.01. (**C**) Chi-square contingency graph of the proportion of counted CD4 T_RMs_ and CD8 T_RMs_ within the CD3 T cell pool of each conjunctival layer expressed as a percentage; *P* < 0.0001; *n* = 7 (except fibrous layer = 6).

To study the distribution of conjunctival CD69^+^ T_RM_ subsets, a panel of antibodies against CD3, CD4, CD69 and HLA-DR was optimised for immunofluorescence microscopy and used on healthy conjunctival sections to enumerate T_RM_ cell subsets. As most CD3^+^ T cells mature to become either CD4^+^ or CD8^+^ single-positive lymphocytes^25^, this study classified CD4 T_RMs_ as CD3^+^ CD4^+^ CD69^+^ HLA-DR^−^ and CD8 T_RMs_ as CD3^+^ CD4^−^ CD69^+^ HLA-DR^−^ (Fig. 2A). We show that both CD4 T_RMs_ and CD8 T_RMs_ are present in all layers of the bulbar conjunctiva, including the deep conjunctiva (see supplementary Tables S2–S5). Specifically, the average number of CD4 T_RM_ cells per mm^2^ was significantly higher in the adenoid layer (292.9 ± 92.2) compared to the superficial epithelium (17.9 ± 3.8; *p* = 0.0034), basal epithelium (61.9 ± 28.0; *p* = 0.0225) and the fibrous layer (28.6 ± 7.6; *p* = 0.0143). When we focused on the average number of CD8 T_RMs_ per mm^2^, we saw no significant difference in the cell count across the conjunctival layers. However, there was a trend for CD8 T_RMs_ to congregate within the basal epithelium (65.1 ± 33.7) with lower cell counts in the superficial epithelium (28.3 ± 13.2), adenoid layer (24.1 ± 10.7) and fibrous layer (9.2 ± 6.0). Within the conjunctival layers, CD4 T_RMs_ predominated over CD8 T_RMs_ in the adenoid layer of the *substantia propria* (292.9 ± 92.2 CD4 T_RMs_ /mm^2^; 24.1 ± 10.7 CD8 T_RMs_/mm^2^; *p* = 0.0024). CD4 T_RM_ and CD8 T_RM_ cell counts per mm^2^ across the superficial epithelium, basal epithelium and fibrous layer showed no significant difference in abundance (Fig. 2B).

### CD69^+^ T_RM_ subsets constitute a large proportion of conjunctival CD3^+^ T cells

CD69^+^ T_RMs_ of the superficial epithelium made up 86% of the total conjunctival CD3^+^ T cell population with CD8 T_RMs_ constituting 53% of CD3^+^ T cells while CD4 T_RMs_ making up the remaining 33% (Fig. 2C). In the basal epithelium, both CD4 T_RMs_ and CD8 T_RMs_ formed 14% of the CD3^+^ T cell population each. We show that within the *substantia propria*, the CD4 T_RM_ population formed the predominate percentage (51% in the adenoid and 29% in the fibrous) of the CD3^+^ T cell population compared to the CD8 T_RMs_ (4% in the adenoid and 9% in the fibrous). Finally, we demonstrate, by means of a Pearson’s Chi-squared test, that there is a significant relation between the tissue layer and the type of CD3^+^ T_RM_ cell subset residing within the layer (*p* < 0.0001) (Fig. 2C).

## Discussion

This study reports the in-situ observation of CD4 and CD8 T_RMs_ in the superficial epithelium, the basal epithelium, the adenoid and the fibrous layers of the healthy human bulbar conjunctiva. To our knowledge, this is the first spatial distribution analysis of T_RMs_ done with complete human conjunctival biopsies. To perform this, we designed and optimised an immunofluorescence panel with antibodies staining against the CD3, CD4, CD69 and HLA-DR surface markers. Using this approach, we demonstrate that human CD4 T_RMs_ and CD8 T_RMs_ are present throughout the entire conjunctival tissue. This confirmed the presence of these T_RMs_ in the superficial epithelium by Bose et al.^23^, where impression cytology was used, as well as identified their presence for the first time in all three deep conjunctival layers: the basal epithelium, the adenoid layer and fibrous layer. As cells of the superficial conjunctival epithelium are continuously sloughed off and shed due to frictional forces from blinking^21,22^, determining the spatial distribution of T_RMs_ in the stable layers of the deep conjunctiva is essential for providing the ocular surface with long-term local immune memory and defence.

CD69^+^ T cells identified using our optimised panel reflect tissue-resident memory T cells that were easily distinguished from circulating memory T cells by their positive CD69 expression^26^. CD69 is an activation marker as well as an inhibitor of sphingosine 1-phosphate receptor-1 that functions to prevent cell egress from tissues^27,28^. HLA-DR is a prominent T-cell activation marker that defines activated T cells as well as myeloid cells including dendritic cells^29,30^. Thus, CD69 detection in the absence of other activation markers such as HLA-DR suggests that CD3^+^ T cells identified using the optimised immunofluorescence panel are CD4 or CD8 T_RMs_. Mean values of CD4 T_RM_ cell numbers per mm^2^ indicated that the majority of these cells congregated abundantly within the adenoid layer of the *substantia propria* just below the basement membrane. T_RMs_ have a distinct profile dependent on specific cytokines including IL (Interleukin)-15, IL-17, IL-33 and transforming growth factor beta (TGF-β) that may be required for their differentiation and continual residency within the tissue^18,31^. Such cytokines are produced by epithelial cells and immune cells, viz lymphocytes and macrophages^16,32–34^. The greater abundance of immune cells in the substantia propria compared to the epithelium^24^, and the close association of the *substantia propria*’s adenoid layer to the basal epithelial cells may explain the larger distribution and establishment of T_RMs_ within the adenoid layer. The presence of CD8 T_RMs_ below the superficial epithelium is important as they may be capable of providing adaptive immune responses, especially antiviral cytokines such as interferon-gamma (IFN-γ)^13^, that prevent the establishment of infection in the epithelium and spread to deeper layers of this tissue.

Within the mucosal epithelia of the body, including the conjunctival epithelium, CD8^+^ T cells are found more abundantly than CD4^+^ T cells; the opposite is true in the subepithelial *lamina propria*^35^. Similarly, CD4 T_RMs_ in the human skin accumulate within the dermis as opposed to CD8 T_RMs_ that are more abundant within the epidermis^24,36^. However, our results demonstrate that conjunctival CD8 T_RMs_ do reside in the *substantia propria*, and are only significantly outnumbered by CD4 T_RMs_ in one layer, the adenoid layer. As the downregulation of the transcription factors Eomesodermin and T-bet is a requirement for CD8 T_RM_ formation^37^, functional analysis on the expression levels of such transcription factors by conjunctival CD8 T_RMs_ may determine if they form a distinct subset of CD8 T_RMs_ that could explain their similar distributions across all conjunctival layers and also their residence within the unique microenvironment of the conjunctival *lamina propria*—the *substantia propria.* CD8 T_RMs_ have been demonstrated to move slowly in the epidermis of the skin, allowing them to target cells of interest^38^. This calls into question whether conjunctival CD8 T_RMs_ are also capable of clearing viral pathogens in infected cells. In other mucosal tissues, T_RMs_ have two antiviral functions: the first is the direct cytotoxicity of infected cells, limited in scope by their relative immobility, and the second is the secretion of antiviral cytokines which diffuse more widely^15^. In general, CD4^+^ T cells secrete antiviral cytokines and act synergistically with CD8^+^ T cells via cognate interactions with local dendritic cells^39^. They also support and maintain the CD8^+^ T cell responses through the production of cytokines such as IL-21 and IFN-γ^40,41^. Therefore, our identification of CD8 and CD4 T_RMs_ in the deep layers of the conjunctiva is vital for understanding the immune response of this tissue. Our findings that T_RMs_ populate the deep conjunctiva denotes that they may be induced in these layers following antigenic challenges administrated to the conjunctival tissue to establish immunogenic memory against specific pathogenic antigens. Thus, T_RMs_ induced in the deep conjunctiva would be able to provide long-term immunity against specific pathogens and, in the case of T_RMs_ of the *substantia propria*, they may be able to communicate with plasma cells and mononuclear phagocytes such as macrophages that are present within this layer^24^ to elicit a rapid, yet vigorous immunogenic response before the pathogen can enter the lymphatics or blood vasculature.

This study is not without its limitations. Notably, the small number of tissues and limited age range call for further studies to be conducted with a larger number of healthy subjects with a focus on the senior age group (80 + years old). Additionally, T_RMs_ in this study were identified based on the consideration that CD69 is acting as a marker of cellular tissue residency rather than an activation marker when a cell is HLA-DR^−^: HLA-DR is considered a late activation marker^42^ while CD69 is an early T cell activation marker, expressed as early as 2–3 h following stimulation^43^. In view of these kinetics, it is possible but unlikely to have included early activated T cells and thus overestimated the number of T_RMs_ counted in the layers of the conjunctiva. It was not possible to incorporate into our IF panel additional activation markers such as CD25, CD71 or Ki67, whose expression levels start to peak around 24–48 h after antigenic challenge^44^ compared to HLA-DR, which is increased even later in the T-cell activation process with its upregulation at 48–72 h after antigenic challenge^42,45^. However, we included the HLA-DR marker rather than earlier activation markers to avoid including CD4^+^ myeloid cells in the T_RM_ cell count. Nevertheless, as all samples were biopsied from healthy participants, inflammatory and activation markers associated with inflammation were not expected to be prominent. Finally, although the *substantia propria* of tarsal conjunctivae demonstrates a larger abundance of immune CD8^+^ T cells than the bulbar conjunctiva^6^, this tissue is more difficult to collect from patients. Further work may reveal a more abundant presence of CD8 T_RM_ subsets in the tarsal conjunctivae; hence, strengthening the incentive to expand research on human ocular mucosal vaccines to induce them. Nevertheless, this work shows that the bulbar conjunctiva alone may prove to be an effective site for T_RM_ induction by conjunctively applied vaccines.

In the present study, we use immunofluorescence microscopy on conjunctival biopsies that preserve their entire histomorphology to determine the distribution of T_RMs_ in the superficial epithelium and the deeper conjunctival layers. We demonstrate that the entire human conjunctiva is protected by an abundant number of both CD4 and CD8 T_RMs_ in all conjunctival layers, with the majority of T_RMs_ congregating in the adenoid layer just below the basement membrane. Additionally, we show a trend for CD4 T_RMs_ to predominate over CD8 T_RMs_ in the *substantia propria*. As increased presence of some T_RM_ subsets may contribute to local ocular inflammation such as in dry eye disease and experimental autoimmune uveoretinitis^23,46^ and were shown to maintain vitiligo disease in a murine model^47^, identifying abnormal levels of conjunctival T_RMs_ based on insight from the normal biological milieu of the tissue will enable their selective targeting and deletion during therapeutic interventions. In relation to mucosal vaccine design, the use of eye drops to target the induction of antigen-specific T_RMS_ within the deep conjunctiva via eye drops may boost the efficacy of prior systemic vaccine regimens. Such cell induction into the mucosa may be possible without the generation of inflammatory responses by the selective recruitment of endogenous antigen-specific T cells using chemokines after parenteral vaccine priming^48^. T_RMs_’ continual conjunctival surveillance and long-lasting immunogenic memory, along with their ability to interact with neighbouring immune cells to secrete cytokines may allow for effective immune mediation against local infections. Additionally, as mucosal sites of the body are functionally connected^7^, immunological activity against pathogens may not only occur in CALT, but also in NALT and BALT; allowing for wide protection across mucosal tissues where pathogen entry is prevalent.

## Materials and methods

### Ethics approval

This study was in strict compliance with the tenets of the Declaration of Helsinki and Australian Regulations concerning the use of human tissues for biomedical research. Experimental protocols within this study were approved by the Western Sydney Local Health District (WSLHD) Human Research Ethics Committee (HREC 2020/ETH02012).

### Inclusion and exclusion criteria

Healthy cataract surgery patients, both men and women at least 18 years old and willing to provide informed consent were enrolled in this study. Patients were excluded from the study if they had any other ophthalmic disorder, additional systemic diseases or if they were pregnant women, Aboriginal or Torres Strait Islanders or from a vulnerable, intellectually, or mentally challenged group.

### Source of conjunctival samples

Human bulbar conjunctival biopsies (n = 7) of approximately one mm^2^ were obtained immediately following surgical excisions performed under local sub-tenon anaesthetic procedures on healthy patients during their routine cataract surgery at Westmead Hospital. Surgical excisions for the purposes of specimen collection were performed at the beginning of the cataract surgeries. All patients provided written consent prior to sample collection. Tissue samples were spread using fine forceps onto an absorbent cellulose filter paper that oriented the specimen so that the superficial surface of the tissue faced upwards. The tissue was allowed to adhere to the paper for 30 s, then was placed immediately to be floating in 5 mL of 4% paraformaldehyde (Electron Microscopy Sciences, PA, USA) in 50 mL collection tubes for 4 h at room temperature in the dark. Tissues were stored in 70% ethanol prior to paraffin-embedding.

### Histological processing

Fixed tissues were processed in an Excelsior ES tissue processor (ThermoFisher Scientific, Runcorn, England, UK) for 4 h. After paraffin embedding in a cross-sectional orientation, 4 μm paraffin sections were cut and collected onto SuperFrost glass slides (ThermoFisher Scientific, MA, USA). Slides were baked for 2 h at 60 °C, deparaffinised in xylene, and rehydrated with decreasing concentrations of ethanol. Some of the serially sectioned slides were stained with haematoxylin and eosin to determine tissue orientation and structure; others were stained with PAS-Mayer’s haematoxylin to determine the distribution of goblet cells and to confirm the identity of the conjunctival tissue. The rest of the slides were stained with fluorochrome-labelled antibodies for immunofluorescence microscopy. Tissue samples excluded from immunofluorescent staining had folded regions, heavily rolled areas, inflamed epithelia defined by polymorph infiltration, missing goblet cells or large areas filled with erythrocytes from ruptured conjunctival capillaries.

### Immunofluorescence staining

Antigen retrieval was performed for 20 min at 95 °C with the Decloaking Chamber NxGen (BioCare Medical, Pacheco, CA, USA) using a pH 9 Borg Decloaker buffer solution (BioCare Medical, Pacheco, CA, USA). Unless otherwise stated, all slides were washed three times for an accumulative 15 min using Tris-buffered saline (TBS; Sigma, T6664) in Coplin glass jars placed on a rotator. Following a TBS wash, sections were encircled with a hydrophobic fixation pen (Dako, CA, USA), and tissue slides were covered with a blocking buffer (0.1% saponin, 1% bovine serum albumin and 10% normal donkey serum diluted in TBS) to be incubated for 30 min at room temperature. Following a TBS wash, sections were incubated with a primary antibody cocktail for 1 h at 37 °C. Abcam antibodies for primary detection include: mouse CD3 (F7.2.38; 1:10 dilution) and rabbit CD4 (EPR6855; AlexaFluor 647; 1:100 dilution), HLA-DR (EPR6148; AlexaFluor 488; 1:100 dilution), and CD69 (EPR21814; 1:100 dilution). Slides were washed in TBS and incubated with anti-rabbit IgG (DyLight 755) and anti-mouse IgG (AlexaFluor 555) secondary antibodies for 30 min at room temperature. Sections were then washed and counterstained with 4’, 6-diamidio-2-phenylindole (DAPI; 1:1000 dilution; Thermo Fisher Scientific) in the dark for 3 min at room temperature. Slides were washed in phosphate-buffered saline (ThermoFisher Scientific, 18912014) and incubated for 3 min with Vector TrueView Autofluorescence Quenching Kit (Vector Laboratories, Burlingame, CA, USA). Slides were finally washed with TBS and rinsed in Milli-Q water (Merck-Millipore, Darmstadt, Germany). Coverslips were mounted to the slides using the Slowfade-Diamond Antifade Mountant (Thermo Fischer Scientific, Waltham, MA, USA). All secondary antibodies were purchased from Invitrogen and were diluted in a 1:400 ratio. Slides were stored in the dark at 4 °C until later immunofluorescent imaging.

For all immunofluorescent staining, the tissues were stained in parallel with the following negative controls: (1) The omission of primary antibodies; (2) The omission of primary and secondary antibodies, leaving only DAPI nuclear staining; (3) The omission of autofluorescence quenching to rule out the possibility of over-quenching and reducing signals from fluorophore-labelled antibodies.

### Immunofluorescence microscopy

Widefield imaging of the entire conjunctival tissue samples was performed on the Olympus VS120 Slide Scanner using the VS-ASW imaging software (version 2.9) as a base system. The ORCA-FLASH 4.0 VS: Scientific CMOS sensor was used to capture all images under the × 20 objective lens (UPLSAPO 20X; NA = 0.75). Channels scanned were DAPI (10 ms exposure), FITC (100 ms exposure), TRITC (500 ms exposure), Cy5 (500 ms exposure) and Cy7 (1000 ms exposure). Channels from the sectioned images were converted to 16-bit TIFF format, then merged and analysed using FIJI (ImageJ). The maximum and minimum brightness and contrast were adjusted in all channels to create the final composite image. Conjunctival layers were separated by creating masks that allow for T_RM_ cell counting in each layer using the ImageJ cell-counter plugin. For each donor, three sections were analysed for cell counts per mm^2^ and the results were averaged.

### Statistical analysis

GraphPad Prism 9.1.1 (GraphPad Prism, San Diego, CA, USA) software was used for all statistical analyses and graph plotting. Square root transformation was used to improve the distribution of the data. The normality assumption was met, and the assumption of equal variance was checked by looking at the data’s residual plot. Thus, significance in the number of T_RMs_ between the different layers of the conjunctiva and in the number of CD4 T_RMs_ to CD8 T_RMs_ was determined by a two-way analysis of variance (ANOVA) test followed by a Tukey HSD multiple comparison test. Additionally, a chi-squared test was performed to determine if there is an overall association between tissue layer and cell type presence. Results are given as the mean ± standard error of the mean (S.E.M.). A value of *P* < 0.05 was considered significant.

## Supplementary Information

Supplementary Tables.

## Data Availability

The datasets generated during and/or analysed during the current study are available from the corresponding author on reasonable request.
